# Diaqua­dichlorido[5-(3-pyridinio)tetra­zolato-κ*N* ^2^]copper(II) dihydrate

**DOI:** 10.1107/S1600536809033042

**Published:** 2009-09-09

**Authors:** Bo Wang

**Affiliations:** a Ordered Matter Science Research Center, College of Chemistry and Chemical Engineering, Southeast University, Nanjing 210096, People’s Republic of China

## Abstract

The title compound, [CuCl_2_(C_6_H_5_N_5_)_2_(H_2_O)_2_]·2H_2_O, was synthesized by hydro­thermal reaction of CuCl_2_ with 3-(2*H*-tetra­zol-5-yl)pyridine. The Cu^II^ cation, located on an inversion center, is coordinated by two Cl^−^ ions, two N atoms from two 5-(3-pyridinio)tetra­zolate zwitterions and two O atoms from two water mol­ecules in a distorted octa­hedral geometry. In the crystal, mol­ecules are linked into a two-dimensional sheet parallel to (001) by N—H⋯N, O—H⋯N, O—H⋯O and O—H⋯Cl hydrogen bonds involving the pyridinium N atom, the Cl atoms and the coordinated and free water mol­ecules. The latter are disordered over two positions in a 0.54:0.46 ratio.

## Related literature

For general background to metal-organic coordination compounds, see: Chen *et al.* (2000 ▶, 2001 ▶); Fu & Xiong (2008 ▶); Fu *et al.* (2007 ▶); Liu *et al.* (1999 ▶); Xie *et al.* (2002 ▶, 2003 ▶); Zhang *et al.* (2001 ▶); Zhao *et al.* (2004 ▶). For related structures, see: Wang *et al.* (2005 ▶); Fu *et al.* (2008 ▶).
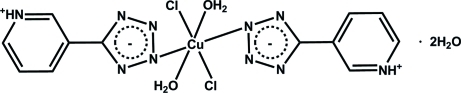


## Experimental

### 

#### Crystal data

[CuCl_2_(C_6_H_5_N_5_)_2_(H_2_O)_2_]·2H_2_O
*M* *_r_* = 500.80
Triclinic, 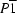

*a* = 6.5484 (13) Å
*b* = 8.3348 (17) Å
*c* = 9.1215 (18) Å
α = 99.54 (3)°
β = 110.22 (3)°
γ = 91.73 (3)°
*V* = 458.64 (19) Å^3^
*Z* = 1
Mo *K*α radiation
μ = 1.53 mm^−1^
*T* = 298 K
0.15 × 0.10 × 0.10 mm

#### Data collection

Rigaku Mercury2 diffractometer
Absorption correction: multi-scan (*CrystalClear*; Rigaku, 2005 ▶) *T*_min_ = 0.85, *T*_max_ = 1.00 (expected range = 0.729–0.858)
4880 measured reflections
2103 independent reflections
1953 reflections with *I* > 2σ(*I*)
*R*_int_ = 0.037

#### Refinement

*R*[*F* ^2^ > 2σ(*F* ^2^)] = 0.045
*wR*(*F* ^2^) = 0.114
*S* = 1.31
2103 reflections
142 parameters
H-atom parameters constrained
Δρ_max_ = 0.33 e Å^−3^
Δρ_min_ = −0.43 e Å^−3^

### 

Data collection: *CrystalClear* (Rigaku, 2005 ▶); cell refinement: *CrystalClear*; data reduction: *CrystalClear*; program(s) used to solve structure: *SHELXS97* (Sheldrick, 2008 ▶); program(s) used to refine structure: *SHELXL97* (Sheldrick, 2008 ▶); molecular graphics: *ORTEPIII* (Burnett & Johnson, 1996 ▶), *ORTEP-3 for Windows* (Farrugia, 1997 ▶) and *SHELXTL* (Sheldrick, 2008 ▶); software used to prepare material for publication: *SHELXTL*.

## Supplementary Material

Crystal structure: contains datablocks I, global. DOI: 10.1107/S1600536809033042/dn2471sup1.cif

Structure factors: contains datablocks I. DOI: 10.1107/S1600536809033042/dn2471Isup2.hkl

Additional supplementary materials:  crystallographic information; 3D view; checkCIF report

## Figures and Tables

**Table 1:** Hydrogen-bond geometry (Å, °)

*D*—H⋯*A*	*D*—H	H⋯*A*	*D*⋯*A*	*D*—H⋯*A*
N1—H1⋯N4^i^	0.86	1.96	2.763 (4)	155
O1*W*—H11*W*⋯O2*WA*	0.85	1.91	2.663 (7)	146
O1*W*—H11*W*⋯O2*WB*	0.85	2.08	2.779 (9)	139
O1*W*—H12*W*⋯Cl1^ii^	0.85	2.42	3.233 (3)	161
O2*WA*—H1*WA*⋯N2	0.85	2.07	2.906 (8)	168
O2*WB*—H1*WB*⋯Cl1^i^	0.85	2.46	3.259 (9)	156

Symmetry codes: (i) 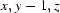; (ii) 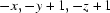.

**Fig. 1.**
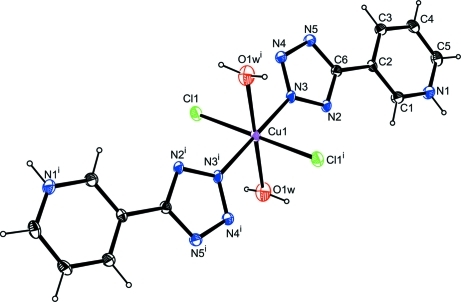
The molecular structure of the title compound, with the atomic numbering scheme. Displacement ellipsoids are drawn at the 30% probability level. H atoms are represented as small sphere of arbitrary radii. Free water molecules have been omitted for clarity. [Symmetry codes: (i) -x+1, -y+1, -z+1].

**Fig. 2.**
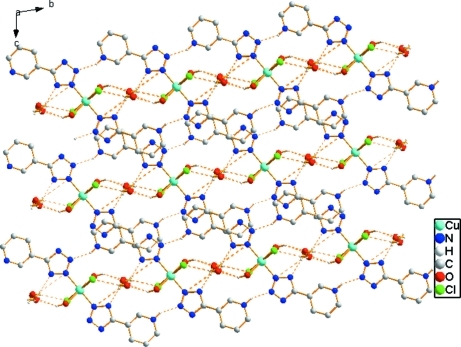
The crystal packing of the title compound, viewed along the c axis, showing the two dimensionnal hydrogen-bonded network. H atoms not involved in hydrogen bonding (dashed line) have been omitted for clarity.

## supplementary crystallographic information

### Comment

The construction of metal-organic coordination compounds has attracted much attention owing to the potential functions, such as permittivity, fluorescence, magnetism and optical properties. (Chen *et al.*, 2000; Chen *et al.*, 2001; Fu *et al.*, 2007; Fu & Xiong 2008; Liu *et al.*, 1999; Xie *et al.*, 2003; Xie *et al.*, 2002; Zhang *et al.*, 2001; Zhao *et al.*, 2004) Tetrazole compounds are a class of excellent ligands for the construction of novel metal-organic frameworks, because of its various coordination modes. (Wang, *et al.* 2005; Fu *et al.*, 2008). We report here the crystal structure of the title compound, Diaqua-dichlorido[pyridinio-3-(2*H*-tetrazolato)-κ*N*] copper(II) dihydrate.

The Cu^II^ cation, located on an inversion center, is coordinated by two Cl^-^ ions, two N atoms from two pyridinio-4-(2H-tetrazolate) zwitterions and two O atoms from two water molecules in a distorted octahedral geometry. The pyridine N atom of the organic ligand is protonated. The pyridinium and tetrazolate rings are essentially coplanar, with a dihedral angle of 0.76 (1)°. The geometric parameters of the tetrazolate ring are comparable to those in related molecules (Wang, *et al.* 2005; Fu *et al.*, 2008).

The molecules are linked into a two-dimensional sheet parallel to the (0 0 1) plane by intermolecular N—H···N, O-H···N, O-H···O and O-H···Cl hydrogen bonds involving the pydine nitrogen and the coordinated and free water molecules. (Table 1 and Fig.2).

### Experimental

A mixture of 3-(2*H*-tetrazol-5-yl)pyridine (0.2 mmol), CuCl_2_ (0.4 mmol), distilled water (1 ml) and a few drops of HCl (6 mol/L) was sealed in a glass tube and maintained at 323 K. Blue block-shaped crystals suitable for X-ray analysis were obtained after 3 d.

### Refinement

All H atoms attached to C atoms and N atom were fixed geometrically and treated as riding with C—H = 0.93 Å and N—H = 0.86 Å with U_iso_(H) = 1.2U_eq_(C or N). H atoms of water molecule were located in difference Fourier maps and included in the subsequent refinement using restraints (O-H= 0.85 (1)Å and H···H= 1.39 (2)Å) with U_iso_(H) = 1.5U_eq_(O). In the last stage of refinement, these H atoms were treated as riding on their parent O atom.

The free water molecule was found to be roughly staistically disoredered over two positions. H atoms for this disordered molecules were treated as above.

### Crystal data

**Table:**

[CuCl_2_(C_6_H_5_N_5_)_2_(H_2_O)_2_]·2H_2_O	*Z* = 1
*M**_r_* = 500.80	*F*(000) = 255
Triclinic, *P*1	*D*_x_ = 1.813 Mg m^−^^3^
Hall symbol: -P 1	Mo *K*α radiation, λ = 0.71073 Å
*a* = 6.5484 (13) Å	Cell parameters from 1953 reflections
*b* = 8.3348 (17) Å	θ = 3.1–27.5°
*c* = 9.1215 (18) Å	µ = 1.53 mm^−^^1^
α = 99.54 (3)°	*T* = 298 K
β = 110.22 (3)°	Block, blue
γ = 91.73 (3)°	0.15 × 0.10 × 0.10 mm
*V* = 458.64 (19) Å^3^	

### Data collection

**Table:**

Rigaku Mercury2 diffractometer	2103 independent reflections
Radiation source: fine-focus sealed tube	1953 reflections with *I* > 2σ(*I*)
graphite	*R*_int_ = 0.037
Detector resolution: 13.6612 pixels mm^-1^	θ_max_ = 27.5°, θ_min_ = 3.1°
CCD profile fitting scans	*h* = −8→8
Absorption correction: multi-scan (*CrystalClear*; Rigaku, 2005)	*k* = −10→10
*T*_min_ = 0.85, *T*_max_ = 1.00	*l* = −11→11
4880 measured reflections	

### Refinement

**Table:**

Refinement on *F*^2^	Primary atom site location: structure-invariant direct methods
Least-squares matrix: full	Secondary atom site location: difference Fourier map
*R*[*F*^2^ > 2σ(*F*^2^)] = 0.045	Hydrogen site location: inferred from neighbouring sites
*wR*(*F*^2^) = 0.114	H-atom parameters constrained
*S* = 1.31	*w* = 1/[σ^2^(*F*_o_^2^) + 1.0933*P*] where *P* = (*F*_o_^2^ + 2*F*_c_^2^)/3
2103 reflections	(Δ/σ)_max_ < 0.001
142 parameters	Δρ_max_ = 0.33 e Å^−^^3^
0 restraints	Δρ_min_ = −0.43 e Å^−^^3^

### Special details

**Table:**

Geometry. All esds (except the esd in the dihedral angle between two l.s. planes) are estimated using the full covariance matrix. The cell esds are taken into account individually in the estimation of esds in distances, angles and torsion angles; correlations between esds in cell parameters are only used when they are defined by crystal symmetry. An approximate (isotropic) treatment of cell esds is used for estimating esds involving l.s. planes.
Refinement. Refinement of F^2^ against ALL reflections. The weighted R-factor wR and goodness of fit S are based on F^2^, conventional R-factors R are based on F, with F set to zero for negative F^2^. The threshold expression of F^2^ > 2sigma(F^2^) is used only for calculating R-factors(gt) etc. and is not relevant to the choice of reflections for refinement. R-factors based on F^2^ are statistically about twice as large as those based on F, and R- factors based on ALL data will be even larger.

### Fractional atomic coordinates and isotropic or equivalent isotropic displacement parameters (Å^2^)

**Table:**

	*x*	*y*	*z*	*U*_iso_*/*U*_eq_	Occ. (<1)
Cu1	0.5000	0.5000	0.5000	0.02259 (18)	
N1	0.2750 (6)	−0.3207 (4)	0.0634 (4)	0.0311 (7)	
H1	0.3009	−0.3948	0.1211	0.037*	
N2	0.3845 (5)	0.1765 (3)	0.2805 (4)	0.0228 (6)	
N3	0.3946 (5)	0.3381 (3)	0.2979 (3)	0.0226 (6)	
N4	0.3283 (5)	0.3805 (4)	0.1585 (4)	0.0259 (7)	
N5	0.2733 (6)	0.2488 (4)	0.0467 (4)	0.0268 (7)	
C1	0.3091 (7)	−0.1653 (5)	0.1350 (5)	0.0294 (8)	
H1A	0.3598	−0.1385	0.2454	0.035*	
C2	0.2700 (6)	−0.0447 (4)	0.0473 (4)	0.0204 (7)	
C3	0.1941 (6)	−0.0902 (4)	−0.1163 (4)	0.0253 (8)	
H3	0.1656	−0.0105	−0.1792	0.030*	
C4	0.1608 (7)	−0.2512 (5)	−0.1860 (5)	0.0299 (8)	
H4	0.1095	−0.2821	−0.2962	0.036*	
C5	0.2037 (7)	−0.3666 (5)	−0.0919 (5)	0.0325 (9)	
H5	0.1825	−0.4770	−0.1375	0.039*	
C6	0.3097 (6)	0.1261 (4)	0.1255 (4)	0.0203 (7)	
O1W	0.3115 (5)	0.3482 (4)	0.6228 (4)	0.0451 (8)	
H11W	0.3277	0.2489	0.6316	0.068*	
H12W	0.1779	0.3657	0.5974	0.068*	
Cl1	0.19420 (15)	0.63743 (11)	0.40795 (11)	0.0307 (2)	
O2WA	0.2969 (13)	0.0279 (8)	0.5219 (8)	0.0506 (16)	0.54
H1WA	0.3360	0.0607	0.4513	0.076*	0.54
H2WA	0.1618	0.0387	0.5026	0.076*	0.54
O2WB	0.1538 (17)	0.0200 (10)	0.5354 (13)	0.068 (3)	0.46
H1WB	0.2070	−0.0699	0.5168	0.102*	0.46
H2WB	0.0279	0.0002	0.5394	0.102*	0.46

### Atomic displacement parameters (Å^2^)

**Table:**

	*U*^11^	*U*^22^	*U*^33^	*U*^12^	*U*^13^	*U*^23^
Cu1	0.0278 (3)	0.0148 (3)	0.0195 (3)	0.0024 (2)	0.0043 (2)	−0.0033 (2)
N1	0.044 (2)	0.0169 (15)	0.0295 (17)	0.0012 (13)	0.0103 (15)	0.0029 (13)
N2	0.0311 (16)	0.0122 (13)	0.0221 (15)	0.0027 (11)	0.0069 (13)	0.0005 (11)
N3	0.0298 (16)	0.0125 (13)	0.0230 (15)	0.0038 (11)	0.0074 (13)	0.0001 (11)
N4	0.0378 (18)	0.0158 (14)	0.0215 (15)	0.0019 (12)	0.0086 (13)	0.0007 (11)
N5	0.0393 (18)	0.0169 (14)	0.0205 (15)	−0.0004 (13)	0.0079 (13)	0.0002 (12)
C1	0.042 (2)	0.0195 (18)	0.0226 (18)	−0.0009 (15)	0.0086 (16)	0.0004 (14)
C2	0.0228 (16)	0.0159 (16)	0.0210 (17)	0.0006 (12)	0.0082 (14)	−0.0017 (13)
C3	0.0301 (19)	0.0221 (18)	0.0217 (18)	0.0010 (14)	0.0078 (15)	0.0017 (14)
C4	0.034 (2)	0.027 (2)	0.0216 (18)	−0.0013 (16)	0.0075 (16)	−0.0072 (15)
C5	0.038 (2)	0.0192 (19)	0.035 (2)	−0.0013 (16)	0.0115 (18)	−0.0071 (16)
C6	0.0215 (16)	0.0168 (16)	0.0204 (17)	−0.0006 (12)	0.0064 (13)	0.0005 (13)
O1W	0.0381 (17)	0.0354 (17)	0.061 (2)	−0.0007 (13)	0.0175 (16)	0.0064 (15)
Cl1	0.0299 (5)	0.0288 (5)	0.0287 (5)	0.0066 (4)	0.0074 (4)	−0.0013 (4)
O2WA	0.069 (5)	0.034 (3)	0.051 (4)	0.008 (3)	0.023 (4)	0.007 (3)
O2WB	0.079 (7)	0.028 (4)	0.109 (8)	−0.001 (4)	0.051 (6)	0.009 (4)

### Geometric parameters (Å, °)

**Table:**

Cu1—N3^i^	1.984 (3)	C2—C3	1.380 (5)
Cu1—N3	1.984 (3)	C2—C6	1.455 (5)
Cu1—Cl1	2.3070 (12)	C3—C4	1.362 (5)
Cu1—Cl1^i^	2.3070 (12)	C3—H3	0.9300
Cu1—O1W	2.390 (3)	C4—C5	1.365 (6)
Cu1—O1W^i^	2.390 (3)	C4—H4	0.9300
N1—C5	1.312 (5)	C5—H5	0.9300
N1—C1	1.325 (5)	O1W—H11W	0.8509
N1—H1	0.8600	O1W—H12W	0.8482
N2—C6	1.314 (4)	O2WA—H1WA	0.8502
N2—N3	1.326 (4)	O2WA—H2WA	0.8506
N3—N4	1.306 (4)	O2WA—H1WB	0.9761
N4—N5	1.313 (4)	O2WB—H2WA	0.3777
N5—C6	1.327 (5)	O2WB—H1WB	0.8518
C1—C2	1.362 (5)	O2WB—H2WB	0.8501
C1—H1A	0.9300		
			
N3^i^—Cu1—N3	180.000 (1)	C2—C1—H1A	119.9
N3^i^—Cu1—Cl1	90.15 (9)	C1—C2—C3	117.9 (3)
N3—Cu1—Cl1	89.85 (9)	C1—C2—C6	120.4 (3)
N3^i^—Cu1—Cl1^i^	89.85 (9)	C3—C2—C6	121.7 (3)
N3—Cu1—Cl1^i^	90.15 (9)	C4—C3—C2	120.3 (4)
Cl1—Cu1—Cl1^i^	180.000 (1)	C4—C3—H3	119.8
N3^i^—Cu1—O1W	87.13 (12)	C2—C3—H3	119.8
N3—Cu1—O1W	92.87 (12)	C3—C4—C5	119.1 (4)
Cl1—Cu1—O1W	89.21 (9)	C3—C4—H4	120.4
Cl1^i^—Cu1—O1W	90.79 (9)	C5—C4—H4	120.4
N3^i^—Cu1—O1W^i^	92.87 (12)	N1—C5—C4	119.6 (3)
N3—Cu1—O1W^i^	87.13 (12)	N1—C5—H5	120.2
Cl1—Cu1—O1W^i^	90.79 (9)	C4—C5—H5	120.2
Cl1^i^—Cu1—O1W^i^	89.21 (9)	N2—C6—N5	112.5 (3)
O1W—Cu1—O1W^i^	180.0	N2—C6—C2	124.4 (3)
C5—N1—C1	122.9 (3)	N5—C6—C2	123.2 (3)
C5—N1—H1	118.6	Cu1—O1W—H11W	124.2
C1—N1—H1	118.6	Cu1—O1W—H12W	112.4
C6—N2—N3	103.8 (3)	H11W—O1W—H12W	110.7
N4—N3—N2	109.9 (3)	H1WA—O2WA—H2WA	109.9
N4—N3—Cu1	122.7 (2)	H1WA—O2WA—H1WB	131.0
N2—N3—Cu1	127.4 (2)	H2WA—O2WA—H1WB	64.2
N3—N4—N5	109.5 (3)	H2WA—O2WB—H1WB	97.6
N4—N5—C6	104.4 (3)	H2WA—O2WB—H2WB	122.2
N1—C1—C2	120.2 (4)	H1WB—O2WB—H2WB	109.3
N1—C1—H1A	119.9		

Symmetry codes: (i) −*x*+1, −*y*+1, −*z*+1.

### Hydrogen-bond geometry (Å, °)

**Table:**

*D*—H···*A*	*D*—H	H···*A*	*D*···*A*	*D*—H···*A*
N1—H1···N4^ii^	0.86	1.96	2.763 (4)	155
O1W—H11W···O2WA	0.85	1.91	2.663 (7)	146
O1W—H11W···O2WB	0.85	2.08	2.779 (9)	139
O1W—H12W···Cl1^iii^	0.85	2.42	3.233 (3)	161
O2WA—H1WA···N2	0.85	2.07	2.906 (8)	168
O2WB—H1WB···Cl1^ii^	0.85	2.46	3.259 (9)	156
O2WB—H2WB···O2WB^iv^	0.85	1.14	1.89 (2)	143

Symmetry codes: (ii) *x*, *y*−1, *z*; (iii) −*x*, −*y*+1, −*z*+1; (iv) −*x*, −*y*, −*z*+1.
